# An Inertial and Optical Sensor Fusion Approach for Six Degree-of-Freedom Pose Estimation

**DOI:** 10.3390/s150716448

**Published:** 2015-07-08

**Authors:** Changyu He, Peter Kazanzides, Hasan Tutkun Sen, Sungmin Kim, Yue Liu

**Affiliations:** 1 Beijing Engineering Research Center of Mixed Reality and Advanced Display, School of Optoelectronics, Beijing Institute of Technology, Beijing 100081, China; E-Mail: wosipo007@163.com; 2 Department of Computer Science, Johns Hopkins University, Baltimore, MD 21218, USA; E-Mails: pkaz@jhu.edu (P.K.); tutkunsen@gmail.com (H.T.S.); sungminkim@jhu.edu (S.K.)

**Keywords:** optical tracking, inertial tracking, hybrid tracking, Extended Kalman Filter

## Abstract

Optical tracking provides relatively high accuracy over a large workspace but requires line-of-sight between the camera and the markers, which may be difficult to maintain in actual applications. In contrast, inertial sensing does not require line-of-sight but is subject to drift, which may cause large cumulative errors, especially during the measurement of position. To handle cases where some or all of the markers are occluded, this paper proposes an inertial and optical sensor fusion approach in which the bias of the inertial sensors is estimated when the optical tracker provides full six degree-of-freedom (6-DOF) pose information. As long as the position of at least one marker can be tracked by the optical system, the 3-DOF position can be combined with the orientation estimated from the inertial measurements to recover the full 6-DOF pose information. When all the markers are occluded, the position tracking relies on the inertial sensors that are bias-corrected by the optical tracking system. Experiments are performed with an augmented reality head-mounted display (ARHMD) that integrates an optical tracking system (OTS) and inertial measurement unit (IMU). Experimental results show that under partial occlusion conditions, the root mean square errors (RMSE) of orientation and position are 0.04° and 0.134 mm, and under total occlusion conditions for 1 s, the orientation and position RMSE are 0.022° and 0.22 mm, respectively. Thus, the proposed sensor fusion approach can provide reliable 6-DOF pose under long-term partial occlusion and short-term total occlusion conditions.

## 1. Introduction

Accurate tracking of the human’s pose is important in augmented reality applications such as entertainment, military training and medical navigation. A number of motion tracking technologies have been developed based on acoustic, mechanical, electromagnetic, optical and inertial sensors [1,2,3,4]. Acoustic systems use either time-of-flight and triangulation or phase-coherence to capture the marker position, but the performance of such devices is seriously affected by the directionality between the transmitters and the receivers. In a mechanical motion tracking system, the ground-based system can only track one rigid body over a small range of motion that is limited by the mechanical structure. An electromagnetic (EM) tracking system tracks the pose of the receiver coil with respect to the EM field generator, which makes the tracking performance suffer from magnetic field distortions when there are ferromagnetic materials in the working volume [5,6]. Optical tracking has been proven to be a reliable and accurate way to capture the pose of a human, but the dependence on markers and cameras makes it only applicable in structured environments and it suffers from occlusion [7]. An inertial tracking system consists of gyros, accelerometers and magnetometers, but is unreliable to track position for long periods of time due to problems with sensor bias and drift [8].

The use of sensor fusion technology is a common approach to compensate the drawbacks of individual tracking methods. The high accuracy of an optical tracking system (OTS) can assist the inertial sensors to remove the bias while the inertial tracking system can improve the robustness of tracking systems by capturing the orientation or position when some of the markers are not visible. Thus, hybrid tracking systems have been developed to take advantage of the complementary benefits of inertial and optical tracking systems. In [9], a hybrid inertial sensor-based indoor pedestrian dead reckoning system aided by computer vision-derived position measurements is proposed. A similar system in [10] consists of a camera and infrared LEDs installed with an inertial measurement unit (IMU) on two shoes to correct inter-shoe position error. In [11], a multi-camera vision system is integrated with a strapdown inertial navigation system to track a hand-held moving device. In most of the existing literatures on tracking technology, variations of a Kalman Filter [12], such as an Extended Kalman Filter (EKF) and Unscented Kalman Filter (UKF), are widely used [5,13,14,15,16,17] to improve the tracking accuracy and robustness. However, line-of-sight is required when the OTS is used to track the motion or improve the tracking performance. When marker occlusion occurs, the absence of vision data, which is widely used as the measurement in Kalman Filter implementations, will result in the failure of position and orientation tracking.

**Figure 1:**
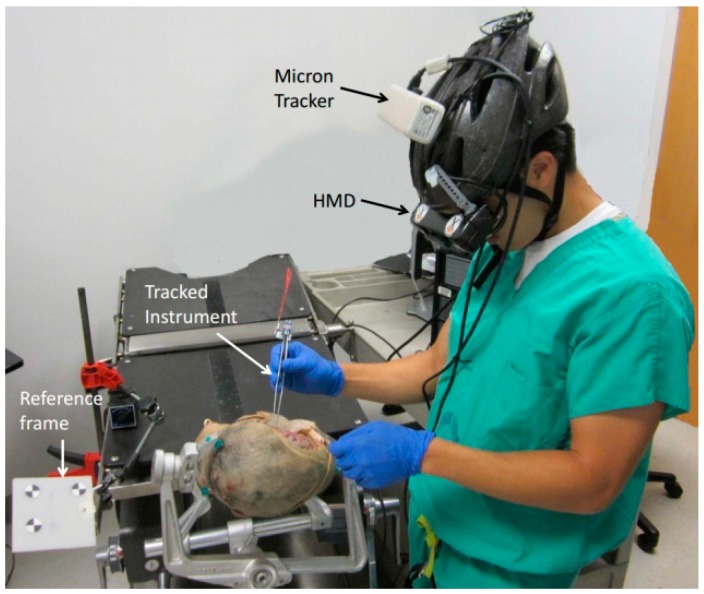
Cadaver experiment with head mounted tracking system and display.

In our previous work [18,19], we proposed a head-mounted optical tracking system for a surgical navigation application, as shown in Figure 1. Optical tracking provides drift-free measurement of position and orientation, but is subject to a line-of-sight constraint and suffers from slower update rates and higher latency [20]. In contrast, inertial sensing, which includes gyroscopes, accelerometers, and magnetometers, provides low latency and high frequency measurement, but these sensors either provide derivatives of position/orientation and are subject to drift, or provide absolute orientation but are subject to bias (e.g., magnetometer) [4,15]. In [21], the authors propose fusion of OTS and IMU measurements to estimate position and orientation in cases of brief occlusions of tracking markers, but only the accelerometer bias is estimated in the EKF. However, in long-term use of the IMU, the accuracy of orientation tracking will be influenced by the biases in the gyroscope and magnetometer. So, in [22] we proposed a sensor fusion approach where the 9-axial measurement from the IMU is bias-corrected by an OTS and used to track the orientation.

In this paper, to reduce the drift error caused by the bias of the inertial sensors and improve the accuracy of 6 degree-of-freedom (DOF) pose tracking, an error model is defined with: the bias errors which are corrected following the bias-correction approach in [22]; and process and measurement noises of the sensor fusion EKF. The camera orientation is used to estimate the bias of the inertial sensors when the full marker frame is visible. Then, an EKF is implemented to estimate the orientation and position with the bias-corrected inertial sensor data as the system state driver and the OTS as part of the measurement when at least one marker is visible. The other part of the measurement is always the orientation from the IMU. Additionally, even when the OTS is capable to track the position, the acceleration measured by the IMU is used to help the hybrid tracking system (HTS) to provide a position tracking result at a higher updating rate.

This paper is organized as follows: Section 2 describes the hybrid tracking system, the error model used to correct the inertial sensor data, and the sensor fusion algorithm applied to track the orientation and position of the target; Section 3 presents the experimental results and discussion; and Section 4 states the conclusions of this paper.

## 2. Methodologies

### 2.1. System Description

**Figure 2:**
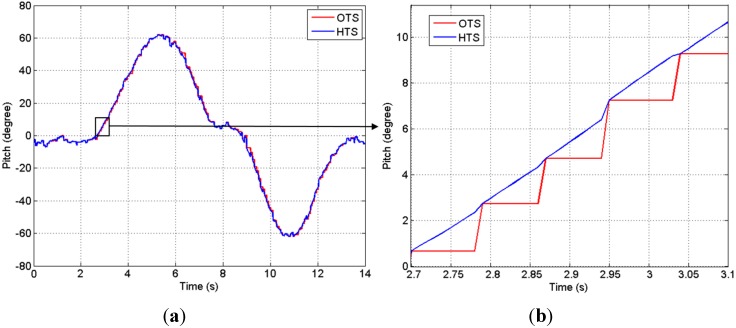
(**a**) Orientation tracking results of the hybrid tracking system (HTS), which has a higher update rate than the optical tracking system (OTS); (**b**) Orientation tracking results in period of 2.7 s–3.1 s. Red: The OTS result at an update rate of 20 Hz. Blue: The synchronized tracking result based on the OTS result and updated by inertial measurement unit (IMU) measurement between two samples of OTS.

The hybrid tracking system (HTS) consists of one stereo camera (Micron Tracker Hx40) as the OTS and one IMU rigidly attached to the camera. The OTS tracks special patterns at approximately 20 fps and a latency of 60 ms, and the captured images are transferred to the host computer via a FireWire port. The IMU contains a 3-axis gyroscope (two-axis IDG300 and a single axis ISZ300 from InvenSense), a 3-axis accelerometer (LIS331DLH from STMicroelectronics) and a 3-axis magnetometer (HMC1043 from Honeywell). It provides the 9 data values of tri-axial accelerometer, gyroscope and magnetometer feedback to the host computer via a USB port at the rate of 100 Hz. The software that captures all sensor data and displays surgical augmented reality (AR) images on the HMD is developed with C++ and implemented on the host PC (MacBook Air). It synchronizes the data at the mean time of sampling from the two tracking units, that is, between the two sampling points of the OTS, the HTS captures the orientation with the IMU measurement, which can supply the tracking data at a higher rate of 100 Hz. As the OTS is assumed to be accurate when the markers are all visible, the synchronized HTS tracking result is based on the slower OTS and updated by the orientation estimated with the IMU measurement as shown in Figure 2.

**Figure 3:**
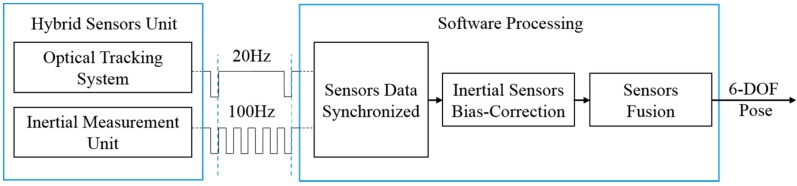
Workflow of the hybrid tracking system.

In order to combine the outputs from OTS and IMU, the two tracking units are registered with a calibration procedure [23] through which the transformation matrix between the two units is achieved. The reference frame consists of three markers attached to a plastic board and is assumed to remain stationary during the procedure (more precisely, all other measurements are made relative to this frame so, without loss of generality, it can be assumed to be stationary). The test setup also includes a surgical instrument that contains three tracked marker points, though in a smaller physical arrangement. The workflow of the hybrid tracking system is shown in Figure 3, which finally outputs the 6-DOF pose including 3-DOF orientation and 3-DOF position. The execution time of the sensor fusion approach is approximately 0.5 ms. Note that our HMD setup combines the IMU with the OTS, but the proposed method would also apply to setups where the IMU is attached to the marker frame(s). The disadvantage of the latter one is the increased complexity of the frame (*i.e.*, IMU electronics that require a power source and wired or wireless communication), but the advantage is that the proposed sensor fusion method can be used for multiple frames.

### 2.2. Inertial Sensors Bias-Correction

The IMU provides the following types of tri-axial measurements: (1) the gyroscope measures the angular rate of rotation; (2) the accelerometer measures the linear acceleration and the acceleration due to gravity; and (3) the magnetometer measures the earth’s magnetic field (*i.e.*, magnetic North).

**Figure 4:**
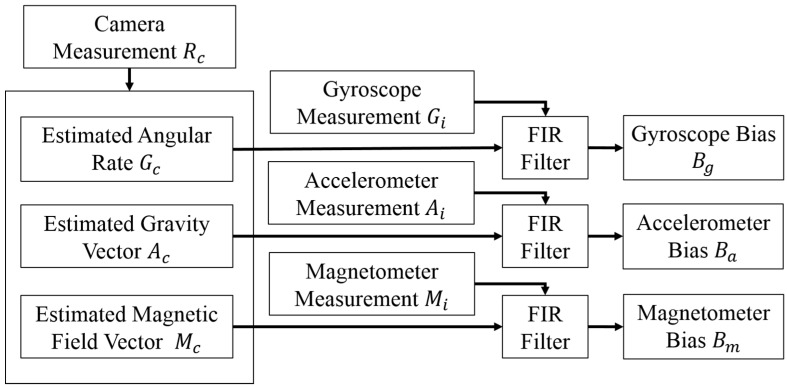
Bias modeling approach.

The position and orientation of objects can be accurately tracked by the OTS, while the marker is in its field of view (FOV). Thus, the output of the optical tracking unit can be used to estimate the bias of the IMU sensors. Specifically, the cosine algorithm is used to calculate the true value of gravity *A_cn_* and magnetic field *M_cn_*, which is orthogonal to the value of gravity from the Euler angles obtained from the camera data. The vector of magnetic field is defined as: X-axis points to the magnetic north; Z-axis is 33.53°orthogonal to the gravity direction; and Y-axis points to the west as right-handed coordinate. The subscripts *i* and *c* indicate measurements from the IMU and camera, respectively. The bias measurement can be estimated by subtracting these true values from the sensor feedback, and then using a Finite Impulse Response (FIR) filter to attenuate the noise, as shown in Figure 4. This approach makes the following simplifying assumptions: (1) there is negligible acceleration related to motion; and (2) the latency between the IMU and optical tracker measurements is negligible. Both of these assumptions are satisfied under quasi-static conditions, where the surgeon’s head is not moving very much.

The FDATool (Filter Design and Analysis Tool, MATLAB) is used to design a low pass FIR filter, for which the sampling frequency is set as 400 Hz, the cutoff frequency is 50 Hz and the order of the FIR filter is 20. The bias model is given by:

(1) $$\left[\begin{array}{c} B_{g}\\ B_{a}\\ B_{m} \end{array}\right]\approx \sum_{n}a_{n}\left[\begin{array}{c} G_{in}-G_{cn}\\ A_{in}-A_{cn}\\ M_{in}-M_{cn} \end{array}\right]$$

where *B_g_*, *B_a_* and *B_m_* are the bias of gyroscope, accelerometer and magnetometer, respectively. *G*, *A* and *M* are the values of angular rate, acceleration and magnetic field, *a_n_* are the coefficients of the FIR filter and satisfy:

(2) $$\sum_{n}a_{n}\text{=}1$$

If the marker points are occluded and there are no orientation results from the OTS, the bias estimation process is stopped and parameters of the bias model are not updated. The most recent bias model is used until the markers become visible again.

### 2.3. Sensor Fusion

#### 2.3.1. Time Updating

We use an EKF to estimate position and orientation from IMU and OTS feedback. The state of the system consists of a unit quaternion *q* that represents the orientation, which is updated by the bias-corrected angular rate, *p* and *v* that represent the position and velocity, which are updated by the bias and gravity corrected linear acceleration. A dynamic state model that describes the evolution of the system state *X_k_*, starting from the system state at the previous step with the function is defined:

(3) $${\hat{X}}_{k}^{-}=f(q,p,v)$$

(4) $$q={\left[\begin{array}{cccc} q_{1} & q_{2} & q_{3} & q_{4} \end{array}\right]}^{T}$$

(5) $$p={\left[\begin{array}{ccc} X & Y & Z \end{array}\right]}^{T}$$

(6) $$v={\left[\begin{array}{ccc} v_{x} & v_{y} & v_{z} \end{array}\right]}^{T}$$

The state is updated by:

(7) $$q_{k+1}=\left[\begin{array}{cccc} 1 & -\frac{\Delta t}{2}(G_{x}-B_{gx}) & -\frac{\Delta t}{2}(G_{y}-B_{gy}) & -\frac{\Delta t}{2}(G_{z}-B_{gz})\\ \frac{\Delta t}{2}(G_{x}-B_{gx}) & 1 & \frac{\Delta t}{2}(G_{z}-B_{gz}) & -\frac{\Delta t}{2}(G_{y}-B_{gy})\\ \frac{\Delta t}{2}(G_{y}-B_{gy}) & -\frac{\Delta t}{2}(G_{z}-B_{gz}) & 1 & \frac{\Delta t}{2}(G_{x}-B_{gx})\\ \frac{\Delta t}{2}(G_{z}-B_{gz}) & \frac{\Delta t}{2}(G_{y}-B_{gy}) & -\frac{\Delta t}{2}(G_{x}-B_{gx}) & 1 \end{array}\right]q_{k}$$

(8) $$p_{k}=p_{k-1}+\Delta t\cdot v_{k-1}+\frac{1}{2}\Delta t^{2}\cdot (rot\cdot (A_{k-1}-B_{a})-A_{gk})$$

(9) $$v_{k}=v_{k-1}+\Delta t\cdot (rot\cdot (A_{k-1}-B_{a})-A_{gk})$$

where *G* and *A* are the angular rate and acceleration, which are bias-corrected by the bias model *B*. The Kalman *a* *priori* estimate of the error covariance matrix is calculated by:

(10) $$P_{k}^{-}=AP_{k}A^{T}+Q$$

where *Q* is the process noise covariance and is defined in Section 2.4.1, process noise modeling.

The discrete-time matrix *A* is an approximation to the fundamental matrix calculated by taking the Taylor expansion of A(t) around the system dynamics matrix:

(11) $$A={\left[\frac{\partial f(x)}{\partial x}\right]}_{\left(\hat{x}\right)}$$

#### 2.3.2. Measurement Updating

The Kalman Gain *K_k_* is given by:

(12) $${\hat{K}}_{k}=P_{k}^{-}H^{T}{\left(HP_{k}^{-}H^{T}+R\right)}^{-1}$$

where the measurement noise covariance *R* is defined in Section 2.4.2, measurement noise modeling. *P_k_* is the “*a priori*” error covariance, *H* is the Jacobian matrix that relates the measurement to the system state vector. The measurement update is：

(13) $${\hat{X}}_{k}={\hat{X}}_{k}^{-}+{\hat{K}}_{k}(Z_{k}-H{\hat{X}}_{k}^{-})$$

The orientation estimated from the IMU measurement and position estimated from the OTS constitute the measurement model. In this paper, the orientation is always estimated from the bias-corrected measurement by the IMU as mentioned in [24]. When all the marker points are in the FOV, the camera can capture the position of the whole marker frame from the spatial position of the marker points and provide a lower-frequency measurement. The position estimated from the IMU feedback suffers from drifting error in the long term, but is sufficiently accurate over a short time, e.g., 0.1 s, which is the time difference between two OTS measurements. So, in between OTS samples, the EKF relies on the prediction driven by the IMU feedback, which provides a higher update rate of position tracking.

**Figure 5:**
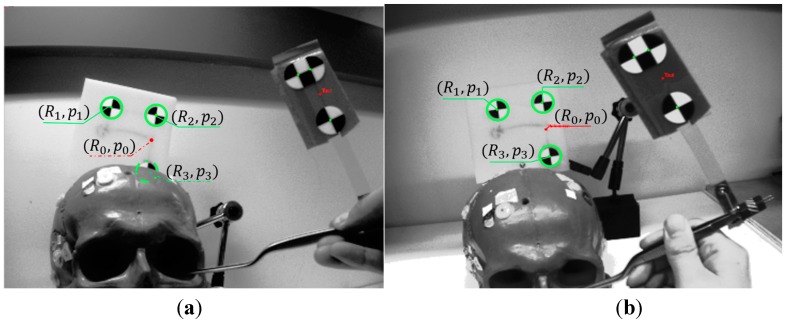
View of the stereo camera. (**a**) Only two marker points are captured during partial occlusion condition; (**b**) All of the marker points are captured without occlusion.

As shown in Figure 5, as soon as any of the marker points is blocked, the OTS cannot give the orientation of the marker frame and the position of the origin (*R_0_*, *P_0_*) (red spot), but it is still possible to obtain the position *P_n_* of any marker that is in the FOV (solid green circle). For the MicronTracker, these stray markers are called XPoints. Other tracking systems, such as Polaris (Northern Digital, Inc., Waterloo, Canada) can also provide the positions of stray markers (*i.e.*, those not associated with a defined rigid body).

Although the position of a stray marker $p_{n}={\left[\begin{array}{ccc} X_{n} & Y_{n} & Z_{n} \end{array}\right]}^{T}$can be obtained, it is necessary to compute the position of the frame origin $p={\left[\begin{array}{ccc} X & Y & Z \end{array}\right]}^{T}$ as the position measurement of the EKF, which requires three pieces of information: (1) identification of marker point (n) to be measured; (2) the distance vector ${\left[\begin{array}{ccc} \Delta X_{n} & \Delta Y_{n} & \Delta Z_{n} \end{array}\right]}^{T}$ from this point to the frame origin; and (3) the orientation of the reference frame:

(14) $${\left[\begin{array}{c} X\\ Y\\ Z \end{array}\right]}_{k}={\left[\begin{array}{c} X_{n}\\ Y_{n}\\ Z_{n} \end{array}\right]}_{k}-\left[\begin{array}{ccc} q_{1}^{2}-q_{2}^{2}-q_{3}^{2}+q_{4}^{2} & 2q_{1}q_{2}+2q_{3}q_{4} & 2q_{1}q_{3}-2q_{2}q_{4}\\ 2q_{1}q_{2}-2q_{3}q_{4} & -q_{0}^{2}+q_{1}^{2}-q_{2}^{2}+q_{3}^{2} & 2q_{2}q_{3}+2q_{1}q_{4}\\ 2q_{1}q_{3}+2q_{2}q_{4} & 2q_{2}q_{3}-2q_{1}q_{4} & -q_{1}^{2}-q_{2}^{2}+q_{3}^{2}+q_{4}^{2} \end{array}\right]\cdot {\left[\begin{array}{c} \Delta X_{n}\\ \Delta Y_{n}\\ \Delta Z_{n} \end{array}\right]}_{k}$$

where q is the quaternion representing the orientation of the reference frame obtained from the EKF. ${\left[\begin{array}{ccc} \Delta X_{n} & \Delta Y_{n} & \Delta Z_{n} \end{array}\right]}^{T}$ is obtained from the marker definition file. The identification of the marker point *n* is realized using a nearest neighbor approach where the position of the marker is compared to the prior estimated positions of all markers, and the closest marker selected. The result of Equation (14) is provided to the EKF as a position measurement during partial occlusion conditions.

**Figure 6:**
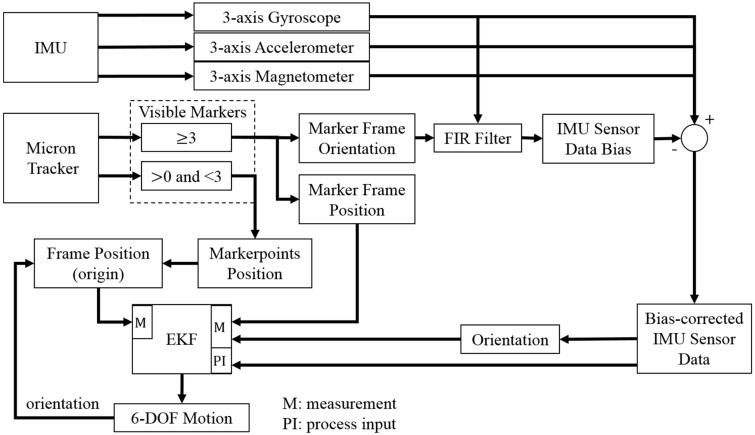
Fusion of inertial sensing to compensate for occlusions in OTS. If there is no occlusion, the MicronTracker can provide the position of the marker frame.

When all the markers are occluded, the optical tracking system cannot provide a measurement. In that case, the system uses the orientation measured by the IMU and relies on the system model to predict the position and velocity based on the IMU acceleration feedback, which is corrected by the estimated bias. As noted previously, the position quickly loses accuracy during full occlusion conditions and can only be trusted for up to about one second. The structure of the sensor fusion approach is shown in Figure 6.

### 2.4. Noise Modeling

#### 2.4.1. Process Noise Modeling

Q is defined as the process noise covariance matrix. In the proposed approach, the continuous process noises of the state are assumed to be independent white noise, thus a normal distribution model in which the mean is zero and the variance σ is established to compute Q:

(15) $$Q=\left[\begin{array}{ccc} {\left[q_{ii}\right]}_{4\times 4} & 0_{4\times 3} & 0_{4\times 3}\\ 0_{3\times 4} & {\left[p_{ii}\right]}_{3\times 3} & 0_{3\times 3}\\ 0_{3\times 4} & 0_{3\times 3} & {\left[v_{ii}\right]}_{3\times 3} \end{array}\right]$$

where *q_ii_* is the process noise of orientation quaternion, *p_ii_* is the process noise of the position vector and *v_ii_* is the process noise of the velocity vector. The process noise parameters are given by:

(16) $$\{\begin{array}{c} q_{ii}\approx N(0,\sigma_{qi}^{2})\\ \begin{array}{l} p_{ii}\approx N(0,\sigma_{pi}^{2})\\ v_{ii}\approx N(0,\sigma_{vi}^{2}) \end{array} \end{array}$$

**Figure 7:**
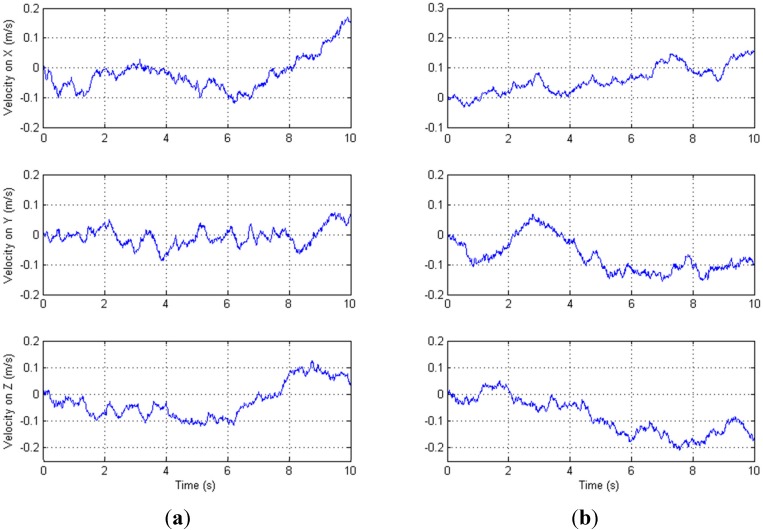
(**a**) Actual velocity; (**b**) Simulated velocity.

To determine the variance that matches the actual process noise, the data from IMU is compared with the data added by simulated noise generated in Matlab in a continuous comparison procedure until a suitable value is obtained. To implement this, the measurement of the IMU is collected for 10 s under static conditions. Figure 7 shows the velocity from the actual accelerometer data (left) and simulated data (right) for the comparison procedure of the velocity process noise. The comparison procedures of the orientation and position process noise are done with a similar method. With the adoption of this comparison procedure, the variance $\sigma_{q}$, which is related to orientation estimation, is set to 0.000025; the variance $\sigma_{p}$, which is related to position estimation, is set to 0.0002; and the variance $\sigma_{v}$, which is related to velocity estimation, is set to 0.00006.

#### 2.4.2. Measurement Noise Modeling

The measurement noise covariance R represents the level of trust of the measurement. To enable the EKF to track both normal-speed motion and abrupt motion, we divide the measurement noise of the orientation quaternion into normal measurement noise covariance *r_a_* and adaptive noise covariance *r_b_*:

(17) $$R=\left[\begin{array}{cc} {\left[r_{a}+r_{b}\right]}_{4\times 4} & 0_{4\times 3}\\ 0_{3\times 4} & {\left[op_{ii}\right]}_{3\times 3} \end{array}\right]$$

When the OTS is occluded and not capable of tracking the orientation, the measurement noise *r_a_* related to the accelerometer and the magnetometer is defined by the same approach proposed in Section 2.4.1. The variance $\sigma_{bi}$, which is related to measurement of inertial sensors, is set to 0.00001. In our approach, the accelerometer is used to sense the gravity vector. In the ideal case, the quadratic sum of the accelerometer measurements *A_X_*, *A_Y_* and *A_Z_* should be *g*^2^, where g is earth gravity. But, the accelerometer reading is also affected by motion (acceleration). We therefore define our error model as the difference between the quadratic sum and *g*^2^ as [15,23]:

(18) $$\left\{ \begin{array}{l} r_{a}\approx N(0,\sigma_{bi}^{2})\\ r_{b}=\left|{A_{x}}^{2}+{A_{y}}^{2}+{A_{z}}^{2}-g^{2}\right|\\ op_{ii}\approx N(0,\sigma_{opi}^{2}) \end{array}\right.$$

Considering that the position tracked by the OTS is assumed to be accurate, the measurement noise variance $\sigma_{opi}$= 0.00000015 when the OTS is not occluded. When the markers are occluded, the OTS does not provide a position measurement so the position measurement update is skipped.

## 3. Results and Discussion

**Figure 8:**
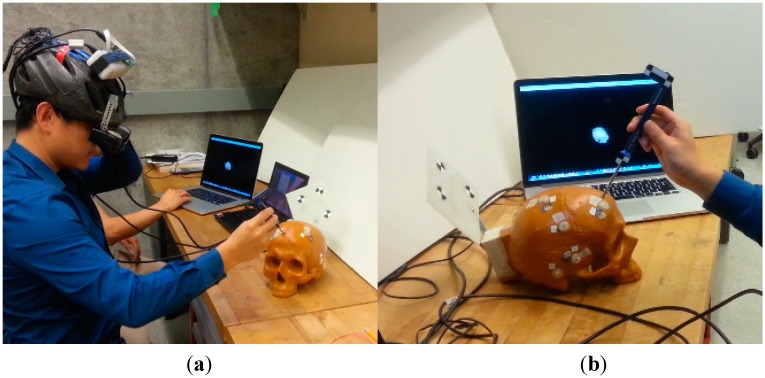
(**a**) Experimental setup; (**b**) Reference marker attached on the surgical target and tool marker attached on the surgical tool.

To validate the sensor fusion method proposed in this paper and evaluate the performance of the orientation and position tracking under partial and total occlusion conditions, experiments are performed with the HTS described in Section 2.1. A skull model attached with the “reference” marker and a surgical tool attached with the “tool” marker are tracked by the HTS, as shown in Figure 8. A similar experimental setup was used in our previous work [18,19], where only the OTS is used to track the markers.

Two sets of motions are used:

Static: To validate the bias calibration algorithm, we keep the HTS and the markers static for 40 min and record the inertial and optical data at an update rate of 100 Hz.

Motion under AR Application: We sequentially rotate the HTS by about 40° around each of the three axes and move the HTS along the three axes for about ±500 mm in the simulated surgical navigation experimental environment.

**Figure 9:**
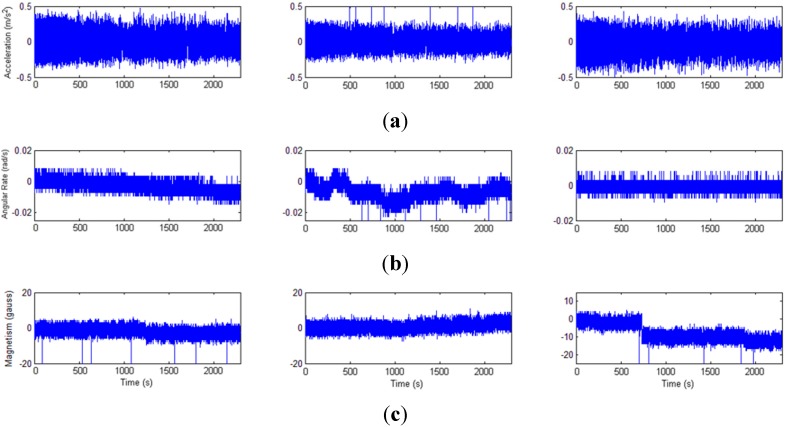
Bias of the inertial sensors. (**a**) Accelerometer; (**b**) Gyroscope; (**c**) Magnetometer.

It can be seen from the static experimental results (Figure 9) that obvious drift of gyroscope and magnetometer data bias exist. Two algorithms with and without the bias correction are compared and the results show that the bias error is voided by using the error model. Table 1 shows that the root mean square errors (RMSE) of the orientation and position tracked by the proposed approach is 43% and 18% less than the result not using the OTS to correct the bias error, respectively.

**Table 1:** Orientation and position tracking results with and without the error model.

	Proposed HTS		HTS without Error Model	
Tests	Orientation RMSE	PositionRMSE	Orientation RMSE	PositionRMSE
1	0.026°	0.101 mm	0.044°	0.129 mm
2	0.023°	0.096 mm	0.039°	0.112 mm
3	0.029°	0.113 mm	0.052°	0.136 mm

**Figure 10:**
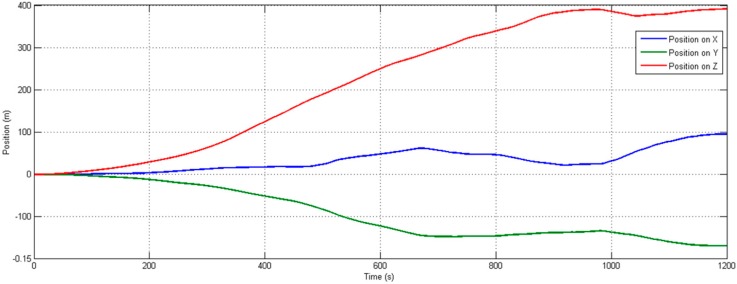
Position (mm) in three-axes *versus* time, obtained by integration of accelerometer data. As expected, results show large errors in position as time increases.

To determine the typical drift rates for the biases and noise of the inertial sensor, an experiment is performed where we compute the orientation and position from the inertial data collected in the static experiment. The orientation is expressed as pitch, roll, and yaw angles and position on the three-axes, and the RMSE is computed by first subtracting the mean value from each set of angles and position. The resulting RMS orientation errors, expressed as pitch, roll, and yaw, are 0.0821°, 0.0495°, and 0.0917°, respectively, which characterizes the orientation error due to both sensor bias drift and noise. Obvious drifting in static position tracking results can be found in Figure 10.

**Figure 11:**
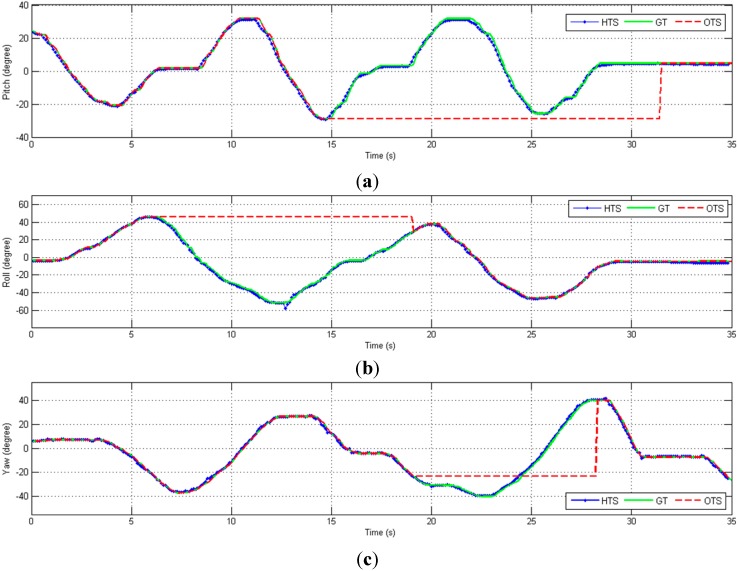
Orientation (degrees) in three-axes *versus* time, including cases of partial OTS occlusion (dashed red lines). (**a**) Pitch; (**b**) Roll; (**c**) Yaw.

To test the dynamic tracking performance under partial occlusion conditions, some of the recorded marker positions are temporarily invalidated. This stops the bias estimation process and relies on the orientation estimated from the IMU measurement to recover the frame origin, as described in Section 2. The orientation tracking result from the hybrid tracking system (HTS) and optical tracking system (OTS) is compared with ground truth (GT) and the comparison results are shown in Figure 11.

Note that in our system, the orientation is always estimated from the measurements of the IMU, so the only effect of marker occlusion is to stop estimation of the bias terms. As shown in the Figure 11, there is no noticeable impact on the estimated orientation when the markers are blocked because the orientation is computed from the inertial sensor data. Thus, the only effect of marker occlusion is that sensor biases are not compensated by the optical tracking data.

**Figure 12:**
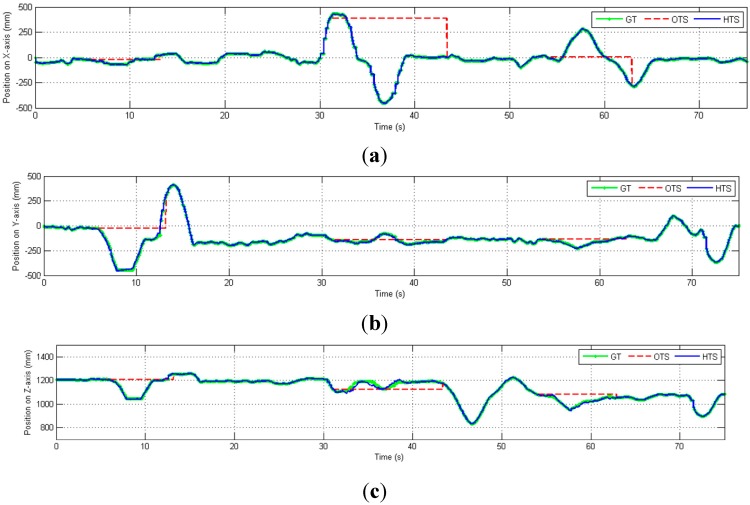
Position (mm) in three-axes *versus* time, including cases of partial OTS occlusion (dashed red lines). (**a**) Position on X-axis; (**b**) Position on Y-axis; (**c**) Position on Z-axis.

We then perform experiments to demonstrate the estimation of the position under partial occlusion conditions. The hybrid tracking system is moved in the X, Y, and Z directions, as shown in Figure 12. The position error due to partial occlusion is relatively small, even though it is affected by inaccuracies in both the position and orientation measurements.

**Figure 13:**
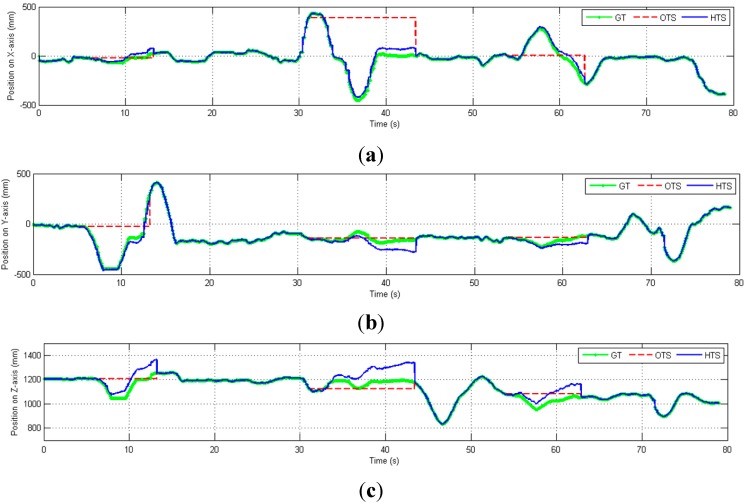
Position (mm) in three-axes *versus* time, including cases of full OTS occlusion (dashed red lines). (**a**) Position on X-axis; (**b**) Position on Y-axis; (**c**) Position on Z-axis.

**Figure 14:**
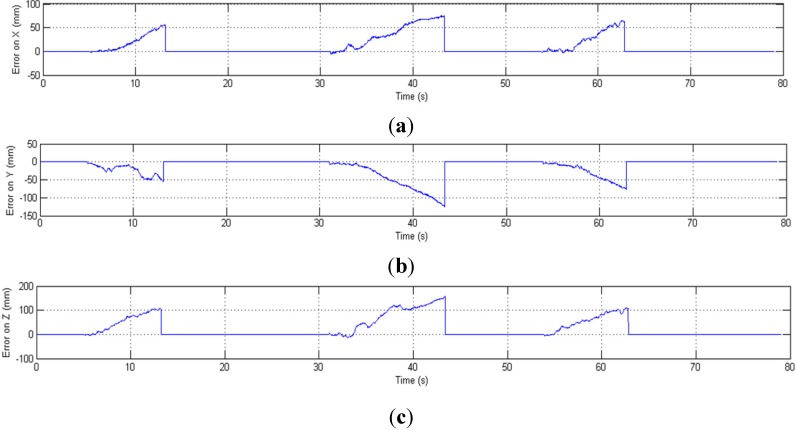
Position error (mm) in three-axes *versus* time; error increases in cases of full OTS occlusion (times corresponding to dashed red lines in Figure 13). (**a**) Error of position on X-axis; (**b**) Error of position on Y-axis; (**c**) Error of position on Z-axis.

If none of the markers are visible, the position tracking is only based on the inertial sensors data. Without the drift-free marker position information and real-time calibration from the OTS, the inertial sensors’ noise is double integrated which causes the estimated position to drift from the ground truth at an increasing rate, as shown in Figure 13 and Figure 14. When all markers are occluded (during 5.5–13.2 s, 31.07–43.4 s and 53.95–62.88 s), the OTS (red) cannot track the position while the HTS (blue) can track the position correctly only in the beginning of the occlusion but quickly drifts away from the ground truth.

The orientation and position tracking results from motion experiments under different conditions are shown in Table 2 and Table 3, where the updating rate is 100 Hz. The position errors when all markers are occluded increases over time at a high rate, but a similar increase is not observed in Table 2, where only some of the markers are occluded. The maximum position error in one minute is 337.1 mm when all markers are occluded, whereas the maximum error with partially occluded markers is 0.173 mm. This demonstrates that with the sensor fusion approach proposed in this paper and incomplete optical information, the HTS can recover the lost orientation information and obtain accurate 6-DOF pose measurements. If all the optical tracking information is missing, the HTS can only limit the position error to a small value (e.g., 0.42 mm) over a short time (e.g., 1 s). In [21], inertial and optical data are fused to track 6-DOF pose, but the bias of inertial sensors is not corrected by the OTS. Their experimental result shows that the position RMSE for a time interval of 3 s is 7.4 mm when one of the markers is visible and 147.3 mm when all of the markers are occluded. Compared with the results in [21], the position and orientation RMSE in Table 2 and Table 3 are lower, which demonstrates that with the help of the bias-correction approach proposed in Section 2, the HTS can more accurately track the 6-DOF motion.

**Table 2:** Hybrid Tracker performance under partial occlusion condition.

Tests	Orientation RMSE	Orientation Max Error	Position RMSE	Position Max Error
1 s	0.019°	0.055°	0.108 mm	0.140 mm
5 s	0.023°	0.048°	0.071 mm	0.145 mm
10 s	0.028°	0.065°	0.118 mm	0.152 mm
20 s	0.031°	0.069°	0.117 mm	0.154 mm
60 s	0.040°	0.090°	0.134 mm	0.173 mm

**Table 3:** Hybrid Tracker performance under full occlusion condition.

Tests	Orientation RMSE	Orientation Max Error	Position RMSE	Position Max Error
1 s	0.022°	0.052°	0.22 mm	0.42 mm
5 s	0.026°	0.058°	3.85 mm	7.23 mm
10 s	0.021°	0.059°	7.41 mm	12.05 mm
20 s	0.028°	0.061°	16.68 mm	31.56 mm
60 s	0.030°	0.077°	146.63 mm	337.1 mm

## 4. Conclusions

This paper presents a sensor fusion approach that combines an OTS with an IMU. The optical tracking result is used to correct the bias of the inertial sensors when all of the markers are visible. The integration scheme is performed in an EKF where the state vector consists of orientation, position, and velocity. The accelerometer and magnetometer feedback are combined to provide a measurement update of the orientation. The position measurement is obtained from the optical tracker when at least one marker is visible. If some markers are occluded, the optical tracker provides the positions of the visible markers and therefore the marker design geometry, in conjunction with the IMU-estimated orientation, is used to compute the frame position. The bias-corrected inertial sensors are used to track position for a short time (up to a few seconds) when all the markers are occluded. Experimental results show that the sensor fusion approach can accurately estimate the 6-DOF pose for long durations when some of the markers are occluded and for a few seconds when all of the markers are occluded.

In practice, it is expected that this sensor fusion approach will provide satisfactory accuracy over relatively long periods of partial marker occlusion. The determining factors include the stability of the estimated bias terms. If the sensor biases drift, it will be necessary to restore full line-of-sight so that the biases can be re-estimated. This is particularly important for the magnetometer bias, which can have large variations due to magnetic field disturbances.

Future work will include applying this method to track both the reference frame and surgical tool, which can be achieved by integrating another wireless and more lightweight IMU on the surgical tool.
